# Synthetic Applications of Chiral Unsaturated Epoxy Alcohols Prepared by Sharpless Asymmetric Epoxidation

**DOI:** 10.3390/molecules15021041

**Published:** 2010-02-23

**Authors:** Antoni Riera, María Moreno

**Affiliations:** Institute for Research in Biomedicine (IRB) and Department of Organic Chemistry, University of Barcelona, Baldiri Reixac, 10. 08028 Barcelona, Spain

**Keywords:** asymmetric synthesis, sharpless asymmetric epoxidation, epoxide ring-opening, olefin metathesis, ring-closing metathesis

## Abstract

An overview of the synthesis and applications of chiral 2,3-epoxy alcohols containing unsaturated chains is presented. One of the fundamental synthetic routes to these compounds is Sharpless asymmetric epoxidation, which is reliable, highly chemoselective and enables easy prediction of the product enantioselectivity. Thus, unsaturated epoxy alcohols are readily obtained by selective oxidation of the allylic double bond in the presence of other carbon-carbon double or triple bonds. The wide availability of epoxy alcohols with unsaturated chains, the versatility of the epoxy alcohol functionality (e.g. regio- and stereo-selective ring opening; oxidation; and reduction), and the arsenal of established alkene chemistries, make unsaturated epoxy alcohols powerful starting materials for the synthesis of complex targets such as biologically active molecules. The popularization of ring-closing metathesis has further increased their value, making them excellent precursors to cyclic compounds.

## Abbreviations

Ac: acetyl
ACN: acetonitrile
Ac_2_O: acetic anhydride
aq.: aqueous
9-BBN: 9-borabicyclo[3.3.1]nonane
Bn: benzyl
Boc_2_O: di-*tert*-butyl dicarbonate
cat.: catalyst
CuTC: copper(I)-thiophene-2-carboxylate
Cy: cyclohexyl
CM: cross metathesis
DCC: dicyclohexylcarbodiimide
DDQ: 2,3-dichloro-5,6-dicyano-1,4-benzoquinone
de.: diastereomeric excess
DIPT: diisopropyl tartrate
DMAP: 4-dimethylaminopyridine
DME: dimethoxyethane
DMF: dimethylformamide
DMP: Dess-Martin periodinane: 1,1,1-triacetoxy-1,1-dihydro-1,2-benziodoxol-3(1*H*)-one
DMPM: 3,4-dimethoxybenzyl
DMSO: dimethyl sulfoxide
dr: diastereomeric ratio
ee: enantiomeric excess
HMPA: hexamethylphosphoramide
Imid.: imidazole
Mes: 2,4,6-trimethylphenyl
MS: molecular sieves
MsCl: mesityl chloride
NMO: *N*-methyl-morpholine-*N*-oxide
nm: not measured
Pip.: piperidine
PMB: *p*-methoxybenzyl
PSA: *p*-TsOH.H_2_O: *p*-toluenesulfonic acid monohydrate
Pyr.: pyridine
RedAl: sodium bis(2-methoxyethoxy)aluminum hydride
RCM: ring-closing metathesis
SAE: Sharpless asymmetric epoxidation
TASF: tris(dimethylamino)sulfonium difluorotrimethylsilicate
TBAF: tetra-*n*-butylammonium fluoride
TBS: *tert*-butyldimethylsilyl
TBDPS: *tert*-butyldiphenylsilyl
TEMPO: 2,2,6,6-tetramethylpiperidine-1-oxyl
TES: triethylsilyl
Tf: triflate
THF: tetrahydrofuran
TIPS: triisopropylsilyl
TMS: trimethylsilyl
TPAP: tetrapropylammonium perruthenate
TsCl: *m*-toluenesulfonyl chloride

## 1. Introduction

The Sharpless asymmetric epoxidation (SAE) [1,2,3] is among the most powerful enantioselective catalytic reactions available. This well-known reaction comprises enantioselective epoxidation of an allyl alcohol using *tert*-butyl hydroperoxide as oxidant. The catalyst can be easily prepared *in situ* by reacting titanium isopropoxide and a chiral tartrate in dichloromethane. The absolute configuration of the resulting epoxide can be easily predicted using a rule developed by Sharpless: it correlates to the enantiomer of the tartrate used. Moreover, Sharpless asymmetric epoxidation is chemoselective. For example, unsaturated epoxy alcohols can be obtained by selective oxidation of an allylic double bond in compounds containing other carbon-carbon double or triple bonds. 

**Figure 1:**
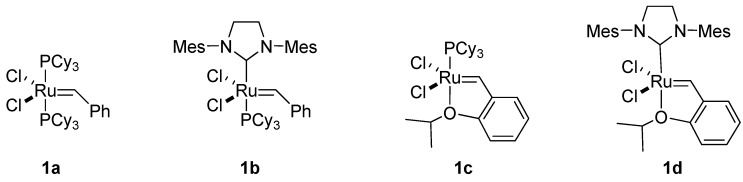
Ruthenium catalysts for metatheses: first-generation Grubbs’ catalyst (**1a**); second- generation Grubbs’ catalyst (**1b**); first-generation Hoveyda-Grubbs’ catalyst (**1c**); and second-generation Hoveyda-Grubbs’ catalyst (**1d**).

Reliable olefin metathesis reactions – chiefly, ring-closing metathesis (RCM) [4,5,6,7] and cross metathesis (CM) [8,9] – have had a major impact on organic synthesis. For example, alkene metatheses have proven invaluable for assembling complex small-molecule targets. Commercialization of the first- and second-generation Grubbs’ catalysts (**1a** and **1b**, respectively; Figure 1), and of the first- and second-generation Hoveyda-Grubb’s catalysts [10,11] (**1c** and **1d**, respectively; Figure 1), has greatly facilitated their use and has fostered synthetic exploitation of chiral unsaturated intermediates, which are excellent precursors for cyclic compounds. Likewise, olefin metatheses have increased the synthetic applications of chiral epoxy alcohols containing olefins in their side chain.

**Figure 2:**
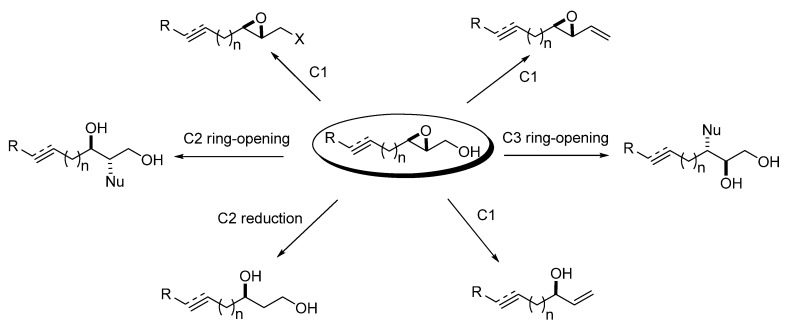
Chemistry of unsaturated 2,3-epoxy alcohols.

The chemistry of the epoxy alcohol fragment in unsaturated epoxy alcohols encompasses the following reactions: a) functional group transformations of the primary hydroxyl group (C1 substitution); b) olefination at C1; c) deoxygenation to an allyl alcohol; d) reduction at C2; and e) epoxide ring-opening at C2 or C3. All of these transformations generate highly functionalized products with excellent regio- and stereo-selectivities (Figure 2).

## 2. Synthesis of Chiral Epoxy Alcohols

There are many examples in the literature of chiral unsaturated epoxides, most of which have been prepared in enantiomerically enriched form by SAE. Table 1 summarizes known epoxy alcohols that contain a double or triple bond less than four carbon atoms away from the epoxide and that were prepared by SAE. As observed, the yields are generally good (except for highly volatile or water-soluble epoxides) and the enantiomeric purities were high (90 to 99% ee).

Although the simplest unsaturated epoxide **2** can be prepared by SAE of 2,4-pentadien-1-ol (**3**), a more convenient route is the kinetic resolution methodology developed by Jagger *et al.* [12,13,14]. This entails SAE of 1,4-pentadien-3-ol (**4**) to give epoxide **5** in excellent enantiomeric excess (>99%), followed by Payne rearrangement [15] of **5** with NaOH 1M to afford **2** (60%). The reason that Jagger’s method provides high enantiomeric excess is because the minor epoxy alcohol formed in the first reaction is removed by epoxidation to the double epoxide; thus, the stereoisomer which forms slower than its homolog, also reacts quicker, generating a double epoxide that can be removed by chromatography.

**Scheme 1:**
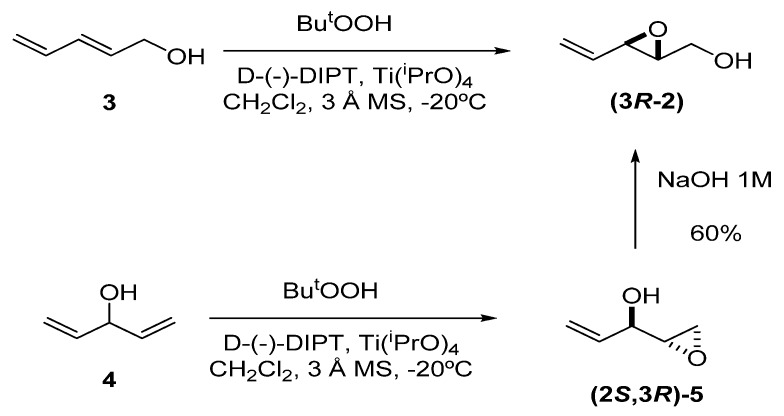
Preparation of epoxy alcohol **2** using either Sharpless asymmetric epoxidation (SAE) or the methodology developed by Jagger, which combines SAE and Payne rearrangement.

**Table 1:** Unsaturated epoxides prepared by Sharpless asymmetric epoxidation.

Epoxy alcohol	Structure	Yield (%)	ee (%)	References
**2**	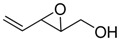	n.d.*	96	[13,57,58,85]
**6**	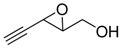	50	nd	[74]
**7**	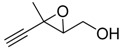	98	61	[53]
**8**	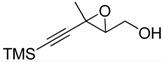	83	88	[52]
**9**	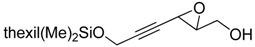	85	95	[51]
**10**	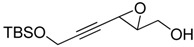	21**	96	[51]
**11**	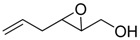	82	92	[82],[47]
***Z* 11**	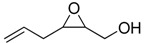	82	>99	[28],[45]
**12**	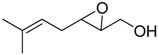	75	93.6	[44]
**13**	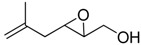	83	93	[88]
**14**	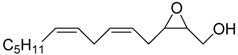	90	nm	[41]
**15**	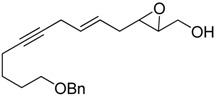	72	nm	[16]
**16**	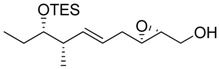	91	90 (de.)	[34]
**17**	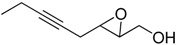	90	nm	[30]
**18**	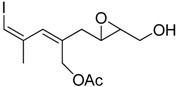	92	>90	[17]
**19**	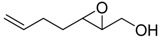	92	94	[82],[68]
**20**	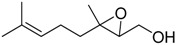	92	91	[18]
**21**	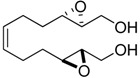	nm	63	[42]
**22**	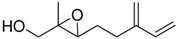	99	92	[19]
**23**	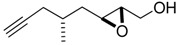	72	98 (de)	[29]
**24**	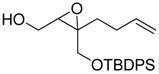	93	97	[26]
**25**	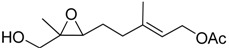	70	>95	[20]
**26**	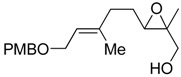	75	99	[37]
**27**	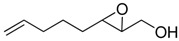	85	91	[82],[25],[50],[49]
**28**	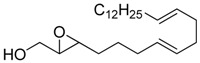	91	>94	[21]
**29**	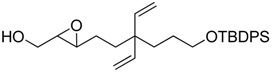	92	>99	[33]

* This compound was not isolated due to its high volatility and reactivity; it was used directly in the subsequent chemistry, without further purification; ** yield for three steps, starting from hexa-2,4-diyne-1,6-diol .

## 3. Transformations of C1

Many of the standard functional group modifications of the primary alcohol (C1) in 2,3-epoxy alcohols [22,23,24] have been used in compounds with unsaturated side chains. The most common of these are oxidation to the aldehyde, halogenation and esterification. The aldehyde offers rich synthetic potential, mainly for nucleophilic attack and olefination. The double bond of the side chain can later be exploited in various ways. 

Before the RCM was developed, one of the classical uses of terminal double bonds was as latent acid derivatives. The synthesis of leukotrienes developed by Spur *et al.* [25] is a prime example: epoxide **(3*S*)-27** (>94% ee) was converted into the epoxy acetate **30** by acetylation followed by oxidation to acid under Sharpless conditions (RuCl_3_, NaIO_4_), and esterification with CH_2_N_2_ in good overall yield. The acetate was readily hydrolyzed in MeOH using a catalytic amount of K_2_CO_3_ plus anhydrous Na_2_SO_4_ (no Payne rearrangement or methyl ester cleavage was observed), providing an epoxy alcohol that was oxidized with Py_2_CrO_3_ to the aldehyde **31**. Crude **31 **was converted into the *trans- *epoxydienal **33 **in 50% yield. Finally, Wittig reaction with the appropriate phosphorane, **34**, gave leukotriene LTA_4_ methyl ester (**35**).

**Scheme 2:**
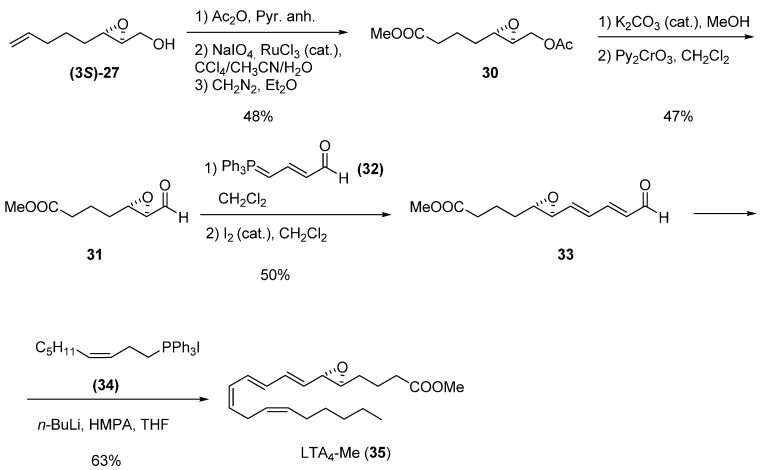
Synthesis of leukotriene LTA_4_ methyl ester (Spur *et al.* [25]).

Among the synthetic advantages of unsaturated epoxides is the ease with which a second point of unsaturation can be introduced into their chain for their subsequent use in RCM. Barrett *et al. *[26] used the enantiomerically enriched (-)-epoxy alcohol **(3*S*)-24 **as starting material in their synthesis of *ent*-Clavilactone B. They oxidized the epoxide under Swern conditions to obtain the (+)-epoxy-aldehyde **36** with 97% ee. As benzyne precursor, they chose 2-fluoro-1,4-dimethoxybenzene (**37**), which upon reaction with *n*-BuLi gave the corresponding o-fluoroaryl lithium species, which in turn was allowed to fragment to a benzyne derivative in the presence of **38**, to afford the aryl Grignard species **39**. Finally, reaction of **39** with epoxy aldehyde **36** gave the two diastereomeric adducts **40**. The crude **40** was converted into a lactone that was then subjected to RCM using the second-generation Grubbs’ catalyst (**1b**). Oxidative demethylation of the resulting adduct gave the target compound **41** with the opposite configuration to the natural clavilactone B.

**Scheme 3:**
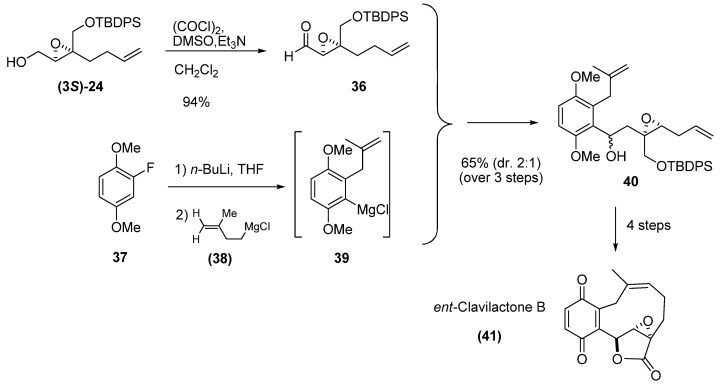
Enantioselective synthesis of *ent*-clavilactone B (Barrett *et al.* [26]).

Another possible transformation at C1 is reduction of the tosylate of the primary alcohol to a methyl group. The tosylate favors the regioselective introduction of an unsaturated chain at C3 by nucleophilic ring-opening of the epoxide. This strategy was used by Martin *et al. *[27] in their synthesis of one of the fragments of the antifungal antibiotic ambruticin S. Tosylation of the epoxy alcohol **(3*S*)-11** furnished epoxide **42**, which was treated with the chiral allyl alcohol **43** in the presence of BF_3_·Et_2_O, to give diene **44**. The tosylate was reduced to the diene **45**, which was then subject to RCM using the first-generation Grubbs’ catalyst (**1a**). Oxidation of the secondary alcohol afforded **46**, one of the pyranyl fragments of (+)-ambruticin S (**47**).

**Scheme 4:**
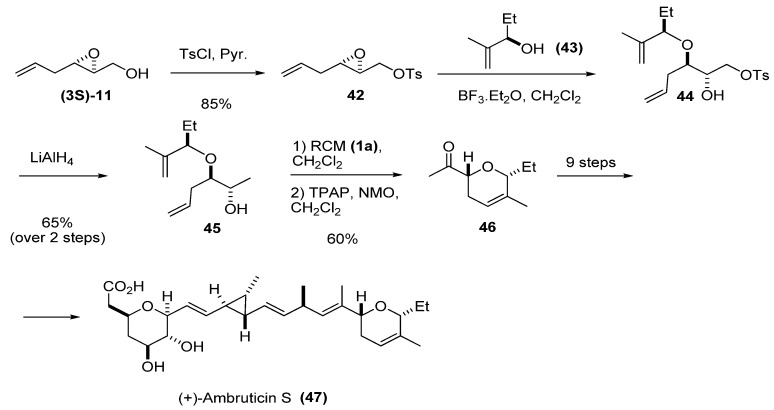
Synthesis of a chiral pyranyl fragment (**46**) of (+)-ambruticin S (**47**) (Martin *et al.* [27]).

**Scheme 5:**
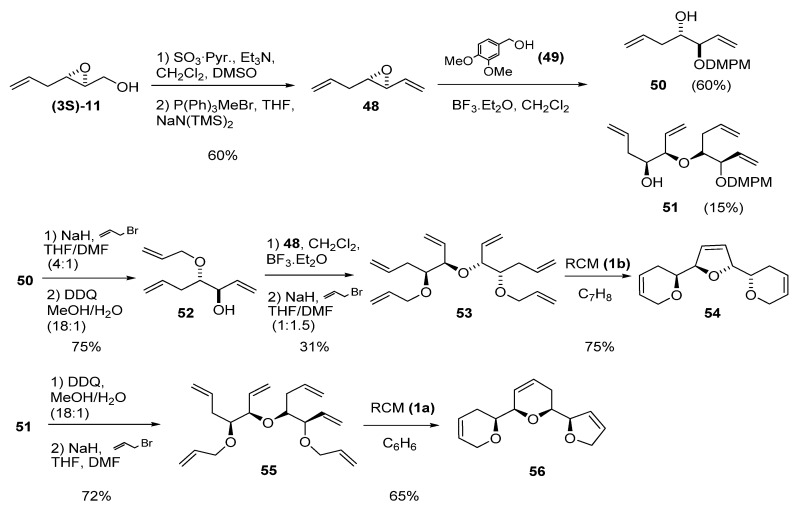
Synthesis of adjacent polycyclic ethers by multiple RCM (Mioskovski *et al.* [28]).

The two-step sequence of oxidation of the primary alcohol to aldehyde followed by Wittig olefination is a useful transformation for introducing a vinylic fragment adjacent to the epoxide. Mioskowski *et al.* [28] combined this methodology with the aforementioned ring-opening with an unsaturated alcohol to prepare polyenes as precursors of tricyclic ethers (found in various natural products) via RCM. Thus, epoxy alcohol **(3*S*)-11** was oxidized to the corresponding aldehyde and submitted to Wittig olefination to yield the vinyl epoxide **48 **(Scheme 5). Epoxide ring-opening of **48** with 3,4-dimethoxybenzyl alcohol (**49**) in CH_2_Cl_2_ gave the expected C2 product **50** plus the alcohol **51** as by-product (formed by ring-opening of **48** with **50**). Alkylation of **50** with allyl bromide followed by deprotection with DDQ afforded the alcohol **52**, which was then used as nucleophile in the ring-opening of **48**. The resulting alcohol was alkylated again with allyl bromide to give the hexaene **53**. A two-step sequence of deprotection followed by alkylation with allyl bromide afforded hexaene **55** from by-product **51. **Both acyclic hexaenes **53 **and **55** were finally submitted to RCM to obtain the triadjacent cyclic ethers **54** and **56**, respectively, in good yields (75 and 65%, respectively).

**Scheme 6:**
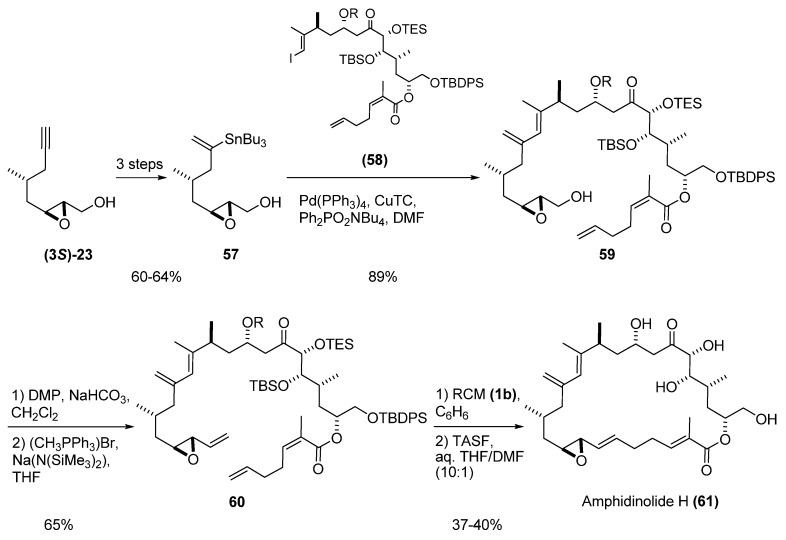
RCM macrocyclization of a vinyl epoxide in the synthesis of amphidinolide H (Fürstner *et al. *[29]).

Fürstner *et al.* [29] used RCM macrocyclization of a vinyl epoxide as the basis of a total synthesis of amphidinolide H (**61**). Thus, the epoxy alcohol **(3*S*)-23** (prepared by SAE) was transformed into the vinyl stannane **57 **(Scheme 6). Cross-coupling reaction with the appropriate vinyl iodide (**58**) afforded compound **59**, which was submitted to the aforementioned sequence of oxidation and Wittig olefination to give the vinyl epoxide **60**. RCM of **60** with the second-generation Grubbs’ catalyst (**1b**) proceeded cleanly, and subsequent deprotection of the silyl protecting groups afforded the target, amphidinolide H (**61**).

**Scheme 7:**
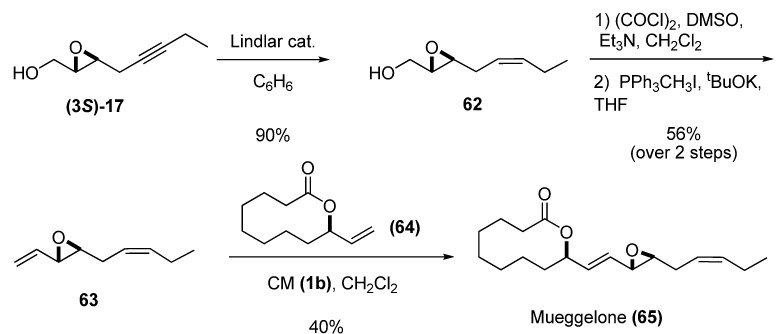
Synthesis of mueggelone based on cross-metathesis (Yadav *et al. *[30])

Vinyl epoxides can also be substrates for cross metathesis (CM), as demonstrated by Yadav *et al.* [30] in their synthesis of the fish-development inhibitor mueggelone from the chiral epoxide **(3*S*)-17** via the sequence shown in Scheme 7. Reduction of **(3*S*)-17** by Lindlar hydrogenation gave the *cis*-olefin **62**, which was subjected to Swern oxidation followed by C1-Wittig homologation [31] to provide the vinyl epoxide **63** in good yield. CM between precursors **63** and **64**, catalyzed by the second-generation Grubbs’ catalyst (**1b**), afforded the target, (+)-mueggelone **(65)**, in 40% yield with complete *E* selectivity.

Reduction of epoxy alcohols to secondary allyl alcohols is a straightforward route to chiral alcohols with fully defined stereochemistry. This transformation can be done using (C_5_H_5_)_2_TiCl, prepared *in situ* from (C_5_H_5_)_2_TiCl_2_ and granulated zinc containing ZnCl_2_, as described by Yadav *et al.* [32]. Interestingly, the chirality of C3 in the epoxide **(3*R*)-17** is totally conserved in the allylic alcohol **66**. 

**Scheme 8:**
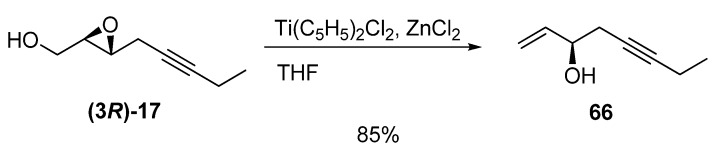
Regioselective deoxygenation of 2,3-epoxy alcohols with (C_5_H_5_)_2_TiCl.

**Scheme 9:**
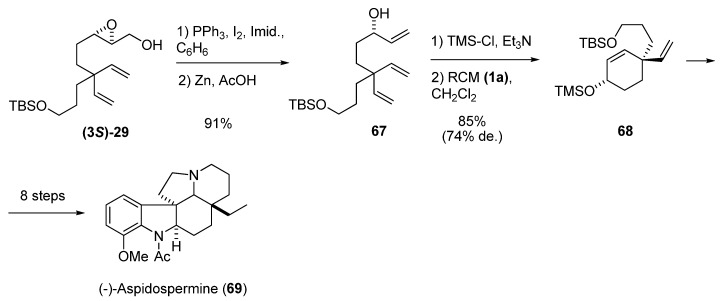
Synthesis of a key intermediate of (-)-Aspidospermine by diastereoselective RCM (Shishido *et al. *[33]).

A similar transformation is reduction of the iodomethyl derivative of the epoxy alcohol. In the synthesis of aspidospermine by Shishido *et al.* [33], the optically pure (> 99% ee) hydroxytriene **67 **was prepared by iodination of the epoxy alcohol **(3*S*)-29** followed by treatment with Zn/AcOH (Scheme 9). After protection of the allyl alcohol, the triene was subjected to diastereoselective RCM to give the cyclohexene **68**, a key intermediate in the synthesis of (-)-aspidospermine (**69**).

**Scheme 10:**
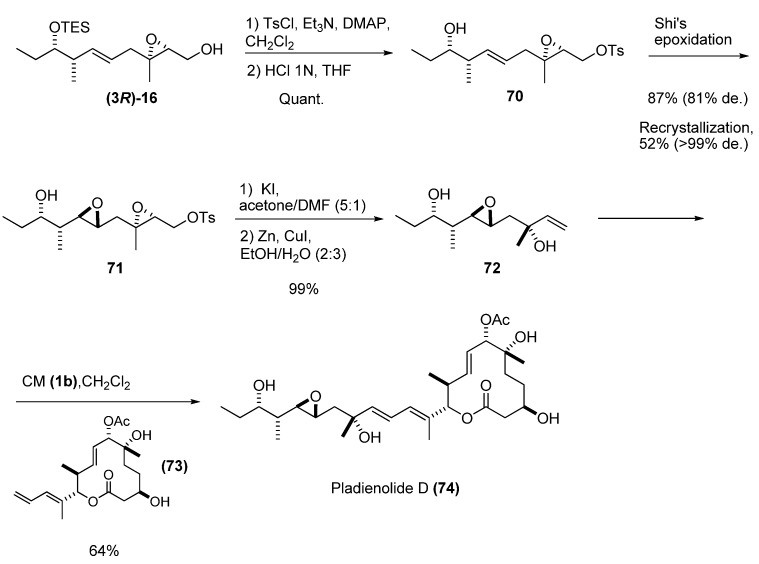
Synthesis of Pladienolide D via cross metathesis of a chiral allylic alcohol (Kotake *et al.* [34]).

Kotake *et al. *[34] also used a chiral allylic alcohol prepared by deoxygenation of an epoxy alcohol to synthesize the potent antitumor compound pladienolide D. The allylic alcohol **72** was prepared from the epoxy alcohol **(3*R*)-16 **(Scheme 10). Tosylation of the primary hydroxyl group and removal of the TES group under weakly acidic conditions provided the alcohol **70**, which was subjected to asymmetric Shi’s epoxidation [35] to the diepoxide **71**. Nucleophilic substitution of the tosylate by iodide and subsequent cleavage of the epoxide with zinc-copper couple [36], gave the desired tertiary allylic alcohol **72 **in excellent yield. Finally, CM with the appropriate olefin fragment (**73**) and the second-generation Grubbs’ catalyst **1b** afforded pladienolide D (**74**) in 64% yield and with excellent stereoselectivity.

**Scheme 11:**
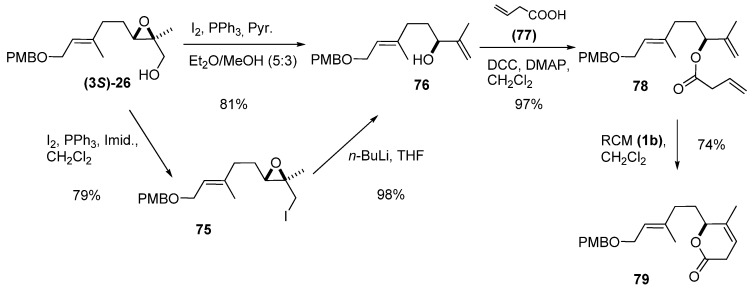
Synthesis of an intermediate in the synthesis of methyl sarcophytoate (Nakata *et al. *[37]*)*.

Nakata *et al. *[37] used the same transformation in their synthesis of a lactone fragment (**79**) (and synthetic precursor) of the marine biscembranoid methyl sarcophytoate (Scheme 11). The enantiomerically enriched epoxy alcohol **(3*S*)-26** was treated with iodine, triphenylphosphine and imidazole to provide epoxy iodide **75**, which was treated with *n*-BuLi to give the allylic alcohol **76**. Alternatively, **76** was directly obtained from **(3*S*)-26** in 81% yield by one-pot treatment of the intermediate iodide with water. To improve its enantiomeric purity, **76** was subjected to kinetic resolution [38], which provided an ee of > 98%. Condensation of **76** with vinyl acetic acid (**77**) using DCC and a catalytic amount of DMAP gave **78**, the substrate for the RCM. Treatment with the second-generation Grubbs’ catalyst (**1b**) afforded the target, **79**, in good yield.

**Scheme 12:**
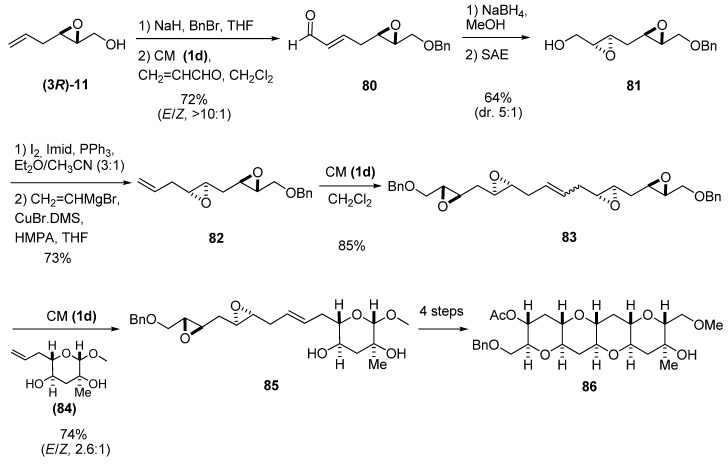
Route to the diepoxide **85**, a synthetic template for the synthesis of a tetracyclic fragment (**86**) of gymnocin A (Jamison *et al. *[39]).

Recently, Jamison *et al. *[39] used epoxide **(3*R*)-11** prepared by SAE as starting material to synthesize compound **86**, a tetracyclic fragment of gymnocin A (Scheme 12). The key step was an epoxide-opening cascade reaction of a triepoxide prepared from **85**. This compound was prepared by CM of alkene **84** and either the diepoxide **82** (synthesized via the sequence shown in Scheme 12) or the self-metathesis product **83**. To this end, compound **(3*R*)-11** was protected as the benzyl ether, and then subject to CM with acroleine using the second-generation Hoveyda-Grubbs’ catalyst **1d** to give the aldehyde **80**. Reduction of **80** to the corresponding alcohol, followed by a second SAE, afforded the diepoxide **81**. Then, a nucleophilic substitution by a vinyl cuprate at C1, gave vinyl diepoxide **82,** which reacted with the second-generation Hoveyda-Grubbs’ catalyst **1d **to give the self-metathesis product **83**. A second CM with the olefin **84** afforded the key intermediate, the diepoxide **85**.

**Scheme 13:**
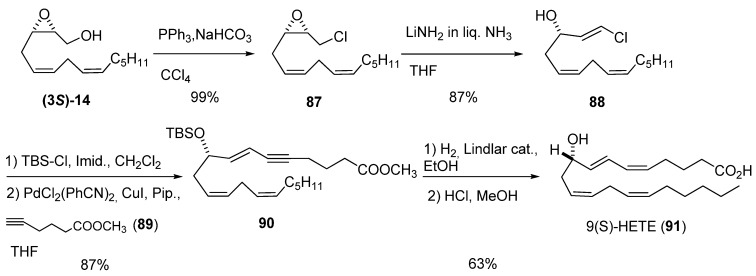
Synthesis of 9(S)-HETE based on preparation of a hydroxy vinyl chloride from an epoxy alcohol (Yadav *et al. *[40]).

Yadav *et al.* [40] developed a methodology to form *trans*-3-hydroxy-vinyl chlorides—which are useful reagents for coupling reactions—by treatment of epoxy chlorides with LiNH_2_. They employed this strategy to prepare both enantiomers of 9-hydroxy eicosatetraenoic acid (HETE) [41]. The epoxy alcohol **(3*S*)-14**, prepared by SAE, was converted into the corresponding chloride **87 **by treatment with PPh_3_ and NaHCO_3_ in refluxing CCl_4 _(Scheme 13). Addition of LiNH_2_ to **87** in liquid NH_3_ at -33 ºC afforded the *trans* vinyl chloride **88** exclusively. After protection of **88 **as the *tert*-butyldimethyl silyl ether, Sonogashira reaction with methyl hex-5-inoate (**89**) afforded the intermediate ester **90**. Finally, **90** was subjected to selective reduction of the triple bond using Lindlar catalyst, followed by deprotection with 1% HCl in MeOH, to obtain the target, **91**. The 9(*R*)-HETE enantiomer was similarly prepared, starting from **(3*R*)-14**.

**Scheme 14:**
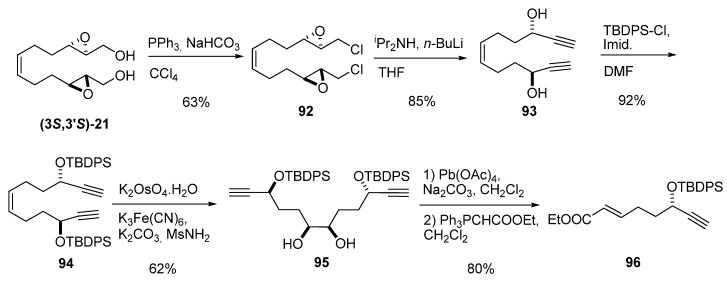
Route to the conjugate ester **96**, a key intermediate in the synthesis of asimin (Marshall *et al.* [42]).

Marshall *et al.* [42] in a modular synthesis of the Annonaceous acetogenin Asimin, prepared the diepoxy diol **(3*S*,*3*’S)-21**, in four steps from 1,5-cyclooctadiene [43], to avoid volatility problems during synthesis of the key intermediate **96 **(Scheme 14). Derivatization of **(3*S*,3’*S*)-21 **to the chloride **92** was followed by elimination to the dialkyne diol **93 **using LDA. Both alcohols were protected as *tert*-butyldiphenyl silyl ethers, and the double bond was dihydroxylated to afford the diol **95**. Oxidative cleavage with lead tetraacetate and subsequent Wittig homologation yielded the conjugate ester **96**.

## 4. Epoxide Ring-Opening at C2

### 4.1. Nucleophilic attack at C2 with carbon nucleophiles

**Scheme 15:**
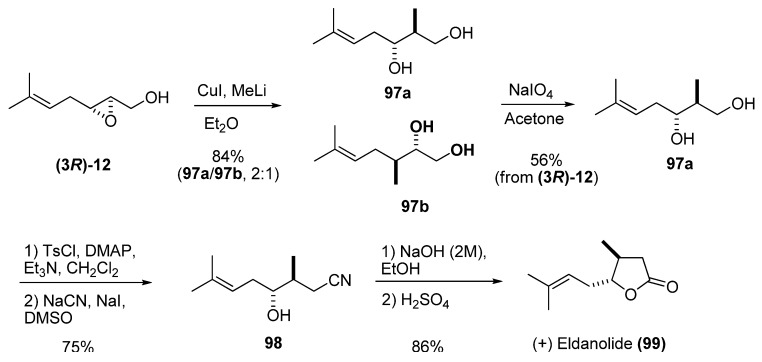
Enantioselective synthesis of (+)-eldanolide (Zhai *et al.* [44]).

Reaction of epoxy alcohols with carbon nucleophiles such as cuprates leads to opening of the epoxide ring. The major isomer is usually the 1,3-diol resulting from the attack at C2. Although the regioselectivity is generally moderate, the minor isomer (the 1,2-diol) can easily be removed by oxidation with sodium periodate. In the synthesis of (+)-eldanolide (**99**), a monoterpenoid pheromone with an unsaturated chain, Zhai *et al.* [44] established the stereochemistry of both stereocenters by opening the unsaturated epoxy alcohol **(3*R*)-12 **with lithium dimethylcuprate, which provided moderate selectivity (Scheme 15). The resulting inseparable mixture of 1,2- and 1,3-diols was treated with NaIO_4_ in acetone to provide the diol **97a** in 56% yield (starting from **(3*R*)-12**). Selective tosylation of **97a** at the primary alcohol followed by S_N_2 displacement gave the nitrile **98** in 75% yield for the two steps. Basic hydrolysis followed by acid-catalyzed lactonization smoothly converted **98** into the desired (+)-eldanolide (**99**).

**Scheme 16:**
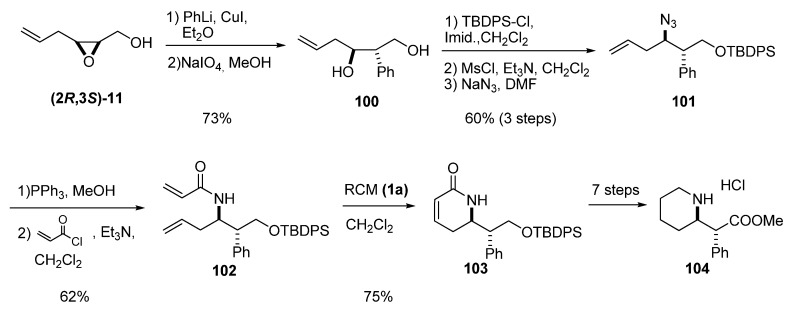
Enantioselective synthesis of *threo*-(+)-methylphenidate hydrochloride (**104**) (Krishna *et al.* [45]).

As mentioned above, the most useful reactivity of the terminal alkenes of the epoxy alcohol’s side chain is in olefinic metatheses. Krishna *et al.* [45], in their synthesis of *threo*-(+)-methylphenidate hydrochloride, treated the epoxy alcohol **(2*R*,3*S*)-11** (from *Z*-hexa-2,5-dien-1-ol) with lithium diphenylcuprate to give a 9:1 mixture of C2/C3 regioisomers (Scheme 16). Once they obtained the desired diol **100**, they protected the primary alcohol and substituted the secondary alcohol with azide to prepare the vinylic azide **101**. Reduction of **101**, followed by treatment of the resulting intermediate with acryloyl chloride, afforded the bisolefin **102**. RCM using the first-generation Grubbs’ catalyst (**1a**) afforded the lactam **103**, which was transformed into the desired (2*R*, 2’*R*)-*threo*-(+)-methylphenidate hydrochloride (**104**). This compound is currently used in racemic form (under the brand name Ritalin) for the treatment of attention deficit hyperactivity disorder (ADHD) in children.

**Scheme 17:**
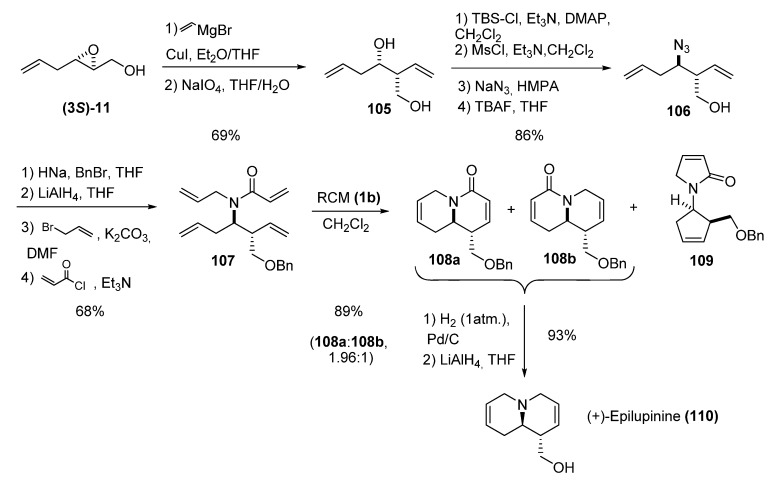
Synthesis of (+)-epilupinine by double RCM (Ma *et al.* [46]).

Ma *et al. *[46] used ring-opening of epoxy alcohols with vinyl cuprate, electrophilic introduction of allyl groups, and double RCM to prepare various isomers of the quinolizidine alkaloid lupinine. Epoxide **(3*S*)-11** was opened by vinyl cuprate to afford the diene **105**, which was subjected to a four-step reaction sequence to generate the azido alcohol **106 **(Scheme 17). Protection of the alcohol followed by reduction of the azide and double alkylation provided compound **107**, the precursor for the double RCM. The RCM afforded three different products: **108a**, **108b** and **109**. Reduction and deprotection of **108a/108b **afforded (+)-epilupinine (**110**) in good yield. The other three isomers of lupinine were likewise synthesized via double RCM reactions of optically active propenamides analogous to **107**.

### 4.2. Nucleophilic reduction at C2 with hydride

**Scheme 18:**
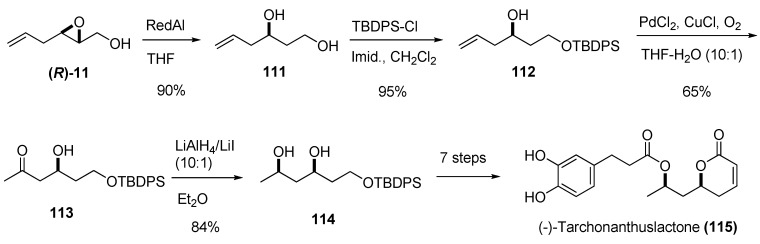
Synthesis of (-)-tarchonanthuslactone (**115**) based on regioselective C2 reduction of the epoxy alcohol **(*R*)-11** (Yadav *et al. *[47]).

Treatment of 2,3-epoxy alcohols with a metal hydride such as Red-Al usually provides regioselective reduction at C2. The corresponding diol is a valuable synthetic intermediate: the two hydroxy groups can be selectively functionalized and the stereochemistry of the secondary alcohol is completely defined. Yadav *et al.* used this strategy to prepare (-)-tarchonanthuslactone (**115**) [47], a pyrone isolated from the leaves of the tree *Tarchonanthus trilobus *(Scheme 18). Reduction of the epoxide **(R)-11** with Red-Al yielded the diol **111**, which was selectively protected at the primary alcohol as the *tert*-butyldiphenyl silyl ether **112**. The terminal double bond of the side chain was converted into the methyl ketone by Wacker oxidation, giving the ß-hydroxy ketone **113** (65%). The key intermediate, the *sy*n-1,3-diol **114**, was conveniently prepared with high diastereoselectivity (*syn*:*anti* selectivity of up to 99:1 or greater) by chelation-controlled reduction of **113** using LiI/LiAlH_4_ [48]. Conversion of **114** into (-)-tarchonanthuslactone (**115**) required seven steps. 

**Scheme 19:**
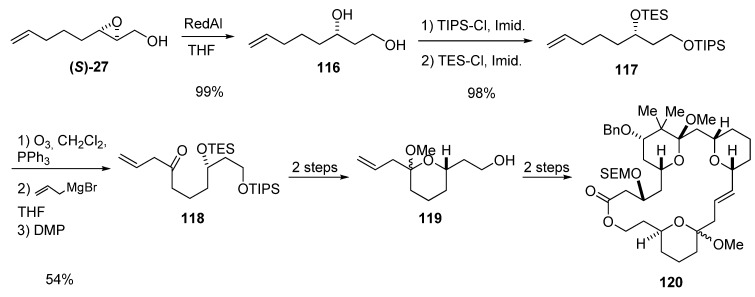
Synthesis of the hemiacetal fragment **119** of the bryostatin analog **120 **(Thomas *et al.* [49]).

Thomas *et al.* [49], in their studies on bryostatin synthesis, constructed the hemiacetal fragment present in these important macrolides from the epoxy alcohol **(*S*)-27 **(Scheme 19). Regioselective reduction of **(*S*)-27** gave the diol **116**, which was selectively protected to give the bis-silyl ether **117**. The terminal alkene was ozonized and the resulting aldehyde treated with allyl magnesium bromide. Oxidation of the resulting alcohol with the Dess-Martin periodinane afforded **118**, which was easily converted into the hemiacetal **119**, the precursor of **120**, which is a macrocyclic bryostatin analog in which the C16-C17 double bond has the (*E*)-configuration.

**Scheme 20:**
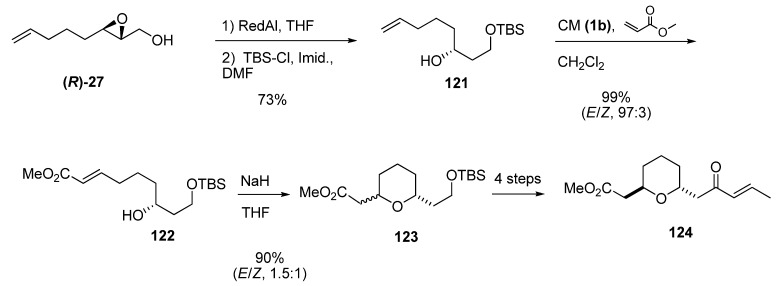
Synthesis of the nor-methyl tetrahydropyran subunit (**124**) of bistramide A (Piva *et al.* [50]).

Piva *et al.* [50] used a similar reduction to synthesize the nor-methyl tetrahydropyran subunit of bistramide A, starting with the enantiomeric epoxide **(*R*)-27 **(Scheme 20). This epoxide was opened by regioselective hydride reduction at C2 with Red-Al, and the primary alcohol of the resulting diol was selectively protected as the *tert*-butyldimethylsilyl ether **121**. CM between **121** and methyl acrylate using the second-generation Grubbs’ catalyst (**1b**) gave the ester **122** (96%). Intramolecular oxa-Michael cyclization of **122** afforded a 1.5:1 mixture of the *trans* and *cis* isomers of the tetrahydropyran **123**. After chromatographic separation and functional group manipulation, the tetrahydropyran ***trans*-124** was obtained in 40% yield (over three steps and a final isomerization) and with 96% ee (as determined by chiral HPLC).

**Scheme 21:**
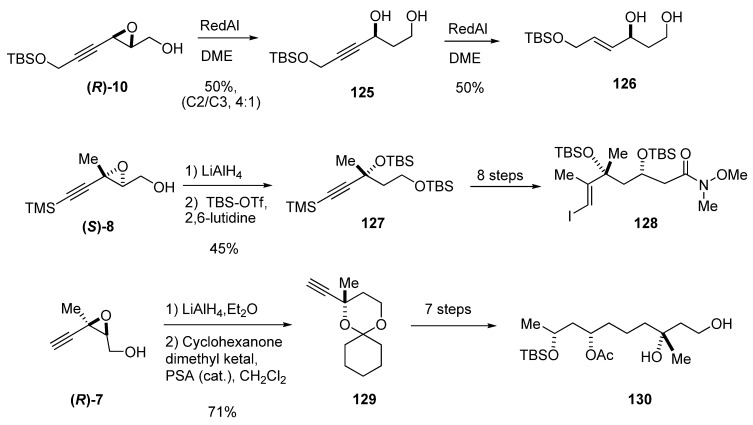
Representative examples of enantioselective syntheses of chiral alcohols by regioselective C2 reduction of alkynyl epoxides.

The regioselective hydride reduction at C2 has also been used in epoxy alcohols with triple bonds in the chain. In 3-alkynyl epoxy alcohols the regioselectivity at C2 is moderate due to the competition from the intramolecular reduction at the activated propargylic position (C3-attack) to give the 1,2-diol. For instance, reduction of **(*R*)-10 **gave the 1,3-diol **125** with a ring-opening selectivity C2/C3 of 4:1 [51]. Diol **125** was further reduced to the *E* allylic alcohol **126** by slow addition of Red-Al, and then purified by chromatography (Scheme 21).

Substitution at C3 further increases the selectivity at C2. Thus, LiAlH_4_ reduction of alkynyl epoxides with a fully substituted C3 center, such as **(*S*)-8** and **(*R*)-7**, is completely regioselective and has proven invaluable for the preparation of chiral tertiary alcohols (Scheme 21). In an asymmetric synthesis of the C_7_-C_20_ synthon of the anti-cancer agent amphidinolide B (**128**), Nelson *et al.* [52] used this methodology to fix the chirality of the tertiary alcohol at C16. Regioselective hydride-mediated oxirane opening of **(*S*)-8** afforded a diol that was then protected with *tert*-butyldimethylsilyl trifluoromethanesulfonate and 2,6-lutidine to give **127**, which was converted into the iodide **128** (subsequently used for coupling) in eight steps (Scheme 21).

Similarly, Ghosh *et al*. [53] efficiently established the chiral center at C3 in Taurospongin A, a non-nucleoside reverse transcriptase inhibitor. The epoxide **(*R*)-7** was reduced with LiAlH_4_ in ether at 0 ºC to the corresponding 1,3-diol which was subsequently treated with cyclohexanone dimethyl ketal using a catalytic amount of *p*-TsOH·H_2_O to provide the ketal **129 **(Scheme 21)**.** Alcohol **130** which had been converted previously to taurospongin A by Lebel and Jacobsen [54] could be prepared from **129** in seven steps.

### 4.3. Nucleophilic attack at C2 with nitrogen nucleophiles

Nitrogen nucleophiles attack epoxy alcohols with low selectivity or with C3 selectivity in the presence of Ti(iOPr)_4 _or LiClO_4 _as Lewis acids. The pioneering work of Roush *et al.* [55,56] enabled directed attack at C2 via intramolecular attack by a carbamic nitrogen. A nitrogen nucleophile can be introduced at C2 with excellent regioselectivity by treating the epoxy alcohol with an isocyanate to form a carbamate that is subsequently cyclized under basic conditions. The selectivity probably stems from kinetic preferential formation of a five-membered ring as compared to a six-membered ring. However, this methodology suffers from a major drawback: certain substrates are prone to isomerize during the basic treatment because of easy acyl transfer from the primary to the secondary alcohol. 

**Scheme 22:**
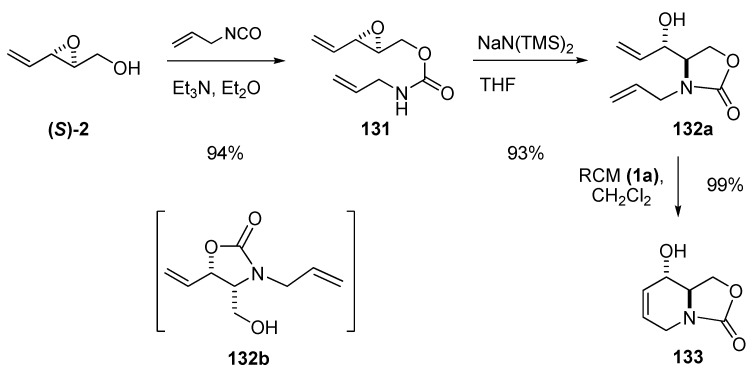
Synthesis of **133**, a key synthetic precursor of several glycosidase inhibitors (Riera *et al. *[57]).

In seeking an efficient enantioselective synthesis of azasugars, Riera *et al.* [57] envisaged preparation of the key intermediate **133** by RCM of the carbamate **132a**, which was prepared from chiral epoxide **(*S*)-2**. The crude epoxide **(*S*)-2** was treated with allyl isocyanate/Et_3_N in refluxing ether to provide allyl carbamate **131** in 94% yield (Scheme 22). However, the subsequent intramolecular ring-opening of **131** under standard conditions (NaH/THF) gave a mixture of the desired oxazolidine **132a** and the *trans*-acetylated isomer **132b**[57]. Other bases (e.g. *tert*-BuOK) gave only slightly better yields, whereas treatment with Lewis acid catalysts such as LiClO_4_ and Ti(iOPr)_4_, led to decomposition of the starting material. Ultimately, sodium bis(trimethylsilyl)amide in THF provided **132a** in 88% yield with no sign of **132b**. Subsequent RCM of **132a** using 5% first-generation Grubbs’ catalyst (**1a**) proceeded smoothly to afford the intermediate **133**. 

**Scheme 23:**
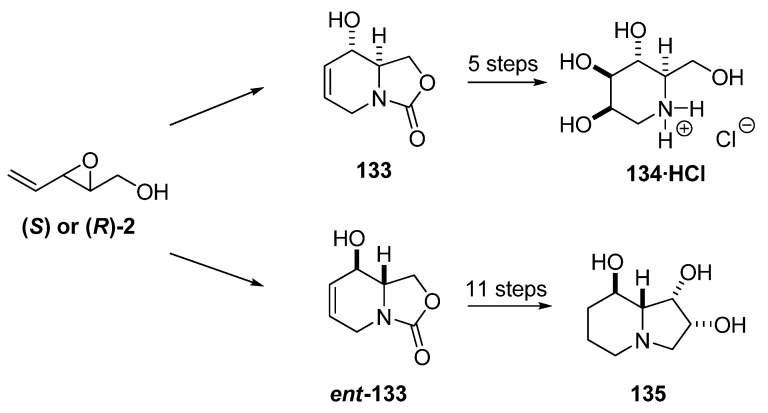
Epoxide **2** as starting material for the synthesis of 1-deoxymannojirimycin (**134**) and swainsonine (**135**) (Riera *et al. *[58]).

The bicyclic carbamate **133** has been converted by Riera *et al.* and others into several glycosidase inhibitors (*e.g.* 1-deoxymannojirimycin (**134**) [58,59,60] and 1-deoxygalactostatin [61]) and other biologically active compounds [62]. Moreover, Riera *et al.* [58] transformed the enantiomeric intermediate ***ent*-133**, prepared from the epoxide (***R*)-2**, into enantiomerically pure swainsonine (**135**), a glycosidase inhibitor with antitumor and antiviral properties (Scheme 23).

**Scheme 24:**
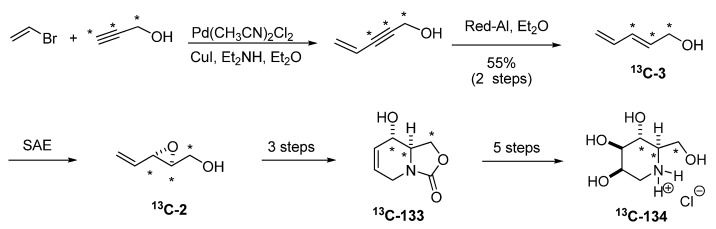
Synthesis of (4,5,6-^13^C)-deoxymannojirimycin (Riera *et al. *[63]).

Riera *et al. *exploited the aforementioned methodology to gain access to a host of ^13^C labeled glycosidase inhibitors for NMR based protein-binding experiments, such as (4,5,6-^13^C)-deoxymannojirimycin [63]. ^13^C labeled (*E*)-2,4-pentadien-1-ol (**^13^C-3**), was prepared from ^13^C-propargyl alcohol by a Sonogashira coupling with allyl bromide followed by reduction (Scheme 24). SAE afforded the isotopically labeled **(*S*)-^13^C-2**, which was run through the sequence shown in Scheme 22, to obtain the isotopically labeled **133,** which was converted into (4,5,6-^13^C)-deoxymannojirimycin (**^13^C-134**). 

**Scheme 25:**
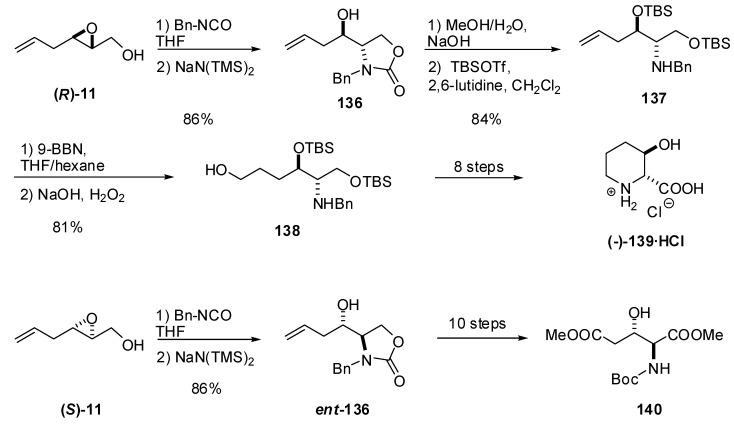
Synthesis of 3-hydroxypipecolic acid hydrochloride (**139**) from the epoxide ***(R)*-11 **and of *erythro*-β-hydroxyglutamic acid methyl ester (**140**) from the epoxide ***(S)*-11 **(Riera *et al.* [64]).

Both enantiomers of benzyl carbamate **136 **can be readily prepared by regioselective C2 ring opening of epoxy alcohols **11 **with benzyl isocyanate (Scheme 25). Riera *et al.* transformed epoxy alcohol **(*R*)-11** into 3-hydroxypipecolic acid (**139**)** [64]**. Hydroboration of the terminal double bond was essential for preparing the alcohol **138**, which, after protecting group manipulation, was cyclized and oxidized to **139**.Analogously, Riera *et al. *used the enantiomer **(*S*)-8** as starting material to synthesize *N*-Boc *erythro*-β-hydroxyglutamic acid methyl ester (**140**) [65]. Oxidation of the terminal double bond to an ethyl ester in an adequately protected derivative of **136** enabled preparation of **140**.

## 5. Epoxide Ring-Opening at C3

### 5.1. Nucleophilic attack at C3 with carbon nucleophiles

**Scheme 26:**
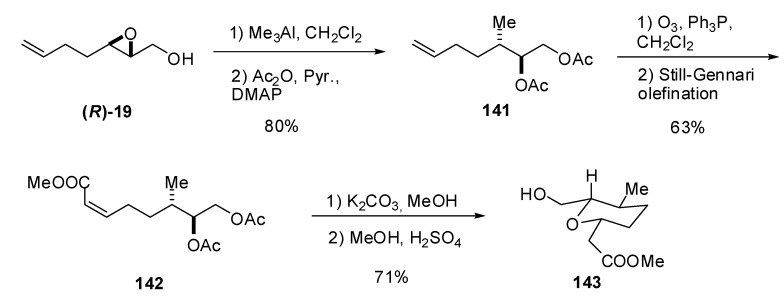
Synthesis of the tetrahydropyranylic core (**142**) of the phytotoxic compound herboxidiene (Banwell *et al.* [68]).

Whereas reaction of epoxy alcohols with cuprates leads to epoxide opening at C2, reaction with trimethylaluminum generally leads to methylation of C3, and consequently, affords a 1,2-diol as the major product. [66,67] Banwell *et al.* [68] exploited this transformation with the unsaturated epoxide **(*R*)-19 **to prepare the tetrahydropyranylic core **143** of the phytotoxic polyketide herboxidiene (Scheme 26). Thus, treatment of **(*R*)-19** with trimethylaluminum provided smooth conversion (opening occurred exclusively on C3) into a diol that was acetylated under standard conditions to obtain **141**. The terminal olefinic bond in **141** was subjected to ozonolytic cleavage, followed by reductive work up with triphenylphosphine, to give an aldehyde that was subjected to a Still-Gennari modification of the Wadsworth-Emmons olefination to afford the unsaturated ester **142**. Finally, hydrolysis of the acetates, followed by acidic treatment, afforded the target **143**. 

In their studies towards the synthesis of briaran diterpenes, Nantz *et al.* [69] reported trimethylaluminum-promoted C3-ring opening of substrates having a fully substituted C3 center.

### 5.2. Nucleophilic attack at C3 with oxygen or sulfur nucleophiles

**Scheme 27:**
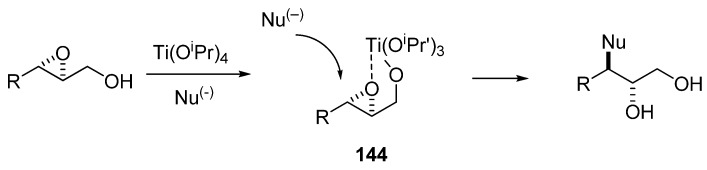
Regioselective C-3 ring-opening of epoxy alcohols under Sharpless conditions.

Sharpless [70] developed the use of Ti(^i^OPr)_4_ to direct the nucleophilic ring-opening of an epoxy alcohol to C3. Computational studies have shown that attack of the intermediate **144** at C3 is kinetically favored (Scheme 27) [71]. The Sharpless conditions are popular because they provide very high regioselectivity. However, nearly the same level of selectivity can be attained using lithium perchlorate in acetonitrile as Lewis acid, as first described by Crotti *et al.* [72]. Although these conditions offer slightly lower regioselectivity, they entail a very simple reaction and work-up.

**Scheme 28:**
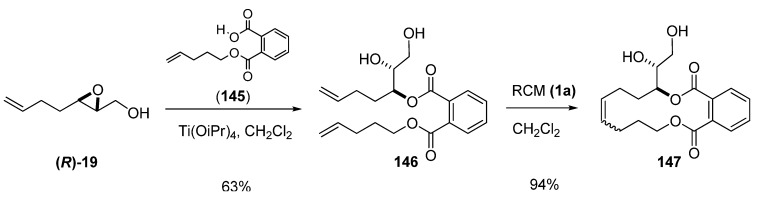
Ring-closing metathesis of a phthalate-tethered diene (Nakata *et al. *[73]).

In the preparation of remotely functionalized compounds, Nakata [73] employed RCM of phthalate-tethered dienes. As a representative example, RCM of diol **146 **using the first-generation Grubbs’ catalyst (**1a**) gave the cyclized diol **147** in 94% yield (Scheme 28)**.** The chiral diene **146** had been prepared from the epoxy alcohol **19** by regioselective epoxide-opening at C3 using a phthalic acid monoester as a nucleophile under Sharpless conditions.

**Scheme 29:**
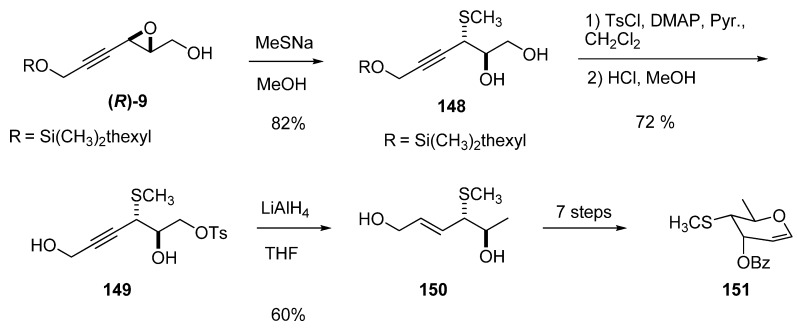
Synthesis of a glycal precursor (**151**) of the trisaccharide found in calicheamycins and esperamycins (Beau *et al. *[51]).

Ring-opening of 3-alkynylepoxy alcohols such as **9** at the propargylic position (C3) by a good nucleophile does not usually require Lewis acid promotion. For example, Beau *et al. *[51] prepared the glycal **151** (a synthetic precursor of the trisaccharide present in calicheamycins and esperamycins) by opening the epoxide **(*R*)-9 **with sodium methanethiolate at 0 ºC in dry MeOH (Scheme 29). The reaction was completely regioselective at C3 to give the diol **148** in 82% yield. The primary hydroxyl group was protected as the tosylate, and then the ether in the resulting adduct was deprotected with acidic MeOH to form the diol **149**. With the correct stereochemistry and substitution at C2 and C3, the C1 position of diol **149** was deoxygenated with concomitant triple bond reduction using LiAlH_4_ in THF at 0 ºC. The stereochemistry of the new double bound was *E*. Finally, the crude diol was recrystallized from toluene to enantiomerically pure **150 **and then transformed into the target glycal **151** in seven steps.

### 5.3. Nucleophilic attack at C3 with nitrogen nucleophiles

**Scheme 30:**
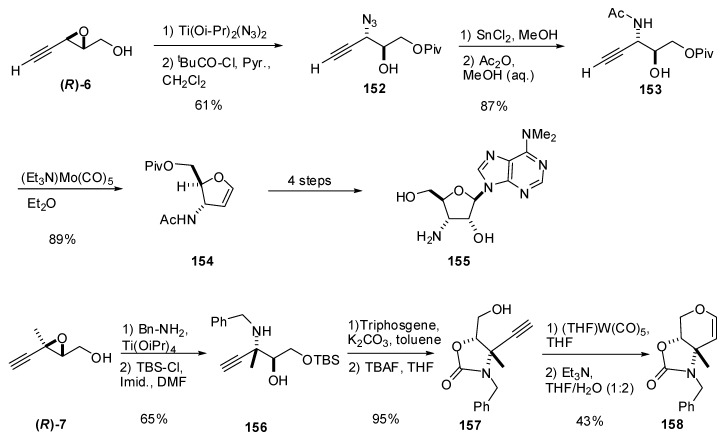
Synthesis of glycals via tungsten or molybdenum carbonyl-induced cyclizations of chiral alkynols (McDonald *et al. *[74]).

In a program devoted to carbohydrate synthesis, McDonald *et al.* [74] studied the preparation of glycals by metal-catalyzed alkynol cyclizations. The starting chiral alkynols were prepared from alkynyl epoxides via C3 ring-opening of alkynyl epoxy alcohols with nitrogen nucleophiles. The epoxide **(*R*)-6** was subjected to titanium-mediated regioselective addition of azide at C3, giving a diol that was then selectively protected at the primary alcohol to give **152 **(Scheme 30). Mosher analysis of the secondary alcohol of **152** indicated an ee of 92%. Reduction of the azide followed by acetylation provided the 3-amidoalkynol **153**, which was submitted to the molybdenum-catalyzed alkynol cyclization to give the 3-amidoglycal **154** (a powerful synthetic precursor to myriad biologically active nucleosides, including puromycin aminonucleoside (**155**)). 

The same research group used the acetylenic epoxide **(*R*)-7** as starting material to prepare the enantiomerically enriched carbamate **157** [75]. This compound was used as a benchmark to study a novel tungsten-carbonyl induced cyclization used to assemble pyranose glycals. Reaction of benzylamine with **(*R*)-7** under Sharpless conditions cleanly opened the epoxide at C3 (Scheme 30). The primary alcohol was then protected as the silyl ether to give **156**. The amine and secondary alcohol were protected as the cyclic carbamate, and the silyl ether was cleaved to afford the alkynyl alcohol **157**, the alkynol substrate for the cyclization. The tungsten carbonyl-induced cyclization of **157** gave a tungsten oxacarbene in good yield, which was converted into the organic pyranose glycal **158** under mildly basic conditions.

**Scheme 31:**
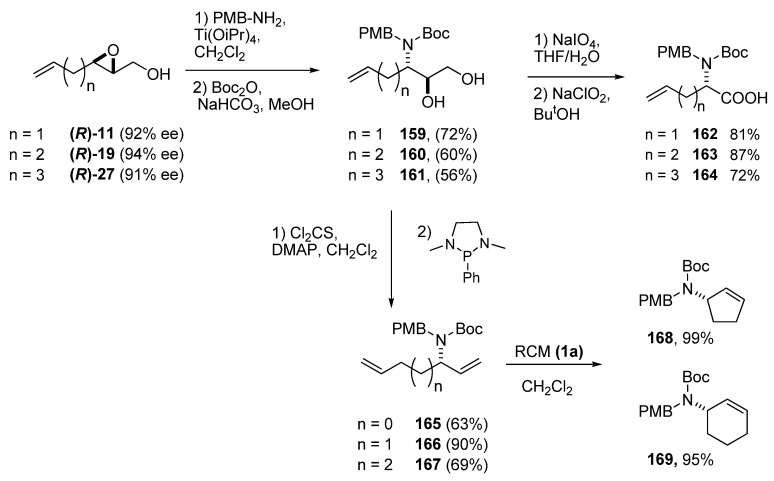
Enantioselective synthesis of unsaturated amino acids and cyclic allyl amines via nucleophilic ring-opening of epoxy alcohols at C3 (Riera *et al.* [76,77,78,79,80,81]).

Riera *et al. *has extensively employed C3 ring-opening of epoxy alcohols with nitrogen nucleophiles [76,77,78,79,80,81] to prepare biologically-active amino acids. Simple, unsaturated amino acids were prepared by using p-methoxybenzylamine as ammonia equivalent [82]. The epoxy alcohols **11, 19** and **27** were treated with p-methoxybenzylamine and Ti(iPrO)_4_, and subsequently protected with Boc_2_O give the *N*-Boc-*N*-(4’-methoxybenzyl)-3-(amino)-1,2-diols **159-161 **(Scheme 31). The 1,2-diol fragments were first cleaved with sodium periodate to the corresponding aldehyde, which was immediately oxidized by sodium chlorite to give the corresponding PMB/Boc protected amino acids **162-164** in good yields. The two protecting groups could be selectively deprotected in any order. Corey-Hopkins deoxygenation of the diol fragment to the terminal alkene [83] enabled preparation of the chiral allylamines **165-167**, which were then submitted to RCM [84]. The cyclopentenyl and cyclohexenyl amines **168** and **169**, respectively, were obtained in excellent yields. However, attempts at cyclizing **165** failed completely (the starting material was completely recovered), most likely due to the strain of the cyclobutane ring.

**Scheme 32:**
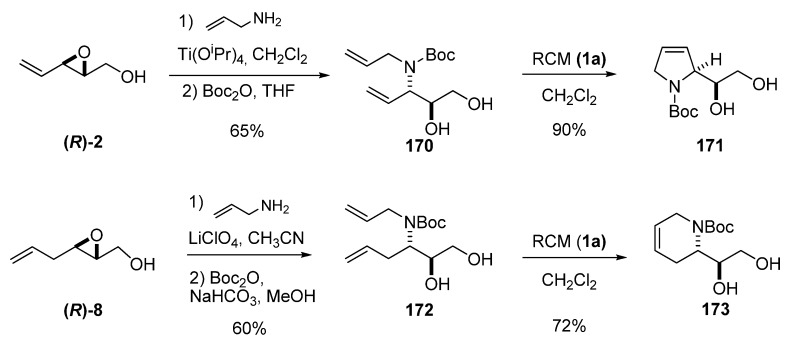
Preparation of dehydropyrrolidine and dehydropiperidine diols (Riera *et al.* [85,86]).

Regioselective and regiospecific ring-opening of unsaturated epoxy alcohols at C3 with allylamine is the most straightforward procedure for introducing the second unsaturation needed to prepare cyclic compounds by RCM. Riera *et al.* [85,86] developed several enantioselective syntheses of biologically interesting compounds based on this methodology. The aminodiol **170 **was prepared via ring-opening of the epoxide **(*R*)-2 **by allyl amine under Sharpless conditions followed by N-Boc-protection in 65% overall yield (Scheme 32). RCM of **170** using the first-generation Grubbs’ catalyst **1a** gave **171** (a key intermediate in the preparation of polyhydroxylated pyrrolidines) in excellent yield. An analogous sequence with the epoxide **(*R*)-8** afforded aminodiol **172**, which was submitted to RCM to give the dehydropiperidine **173** in excellent yield. The ring-opening was performed under Crottis’s conditions using lithium perchlorate. It is worth noting that the key intermediate **173 **was enantiomerically enriched by crystallization. 

**Scheme 33:**
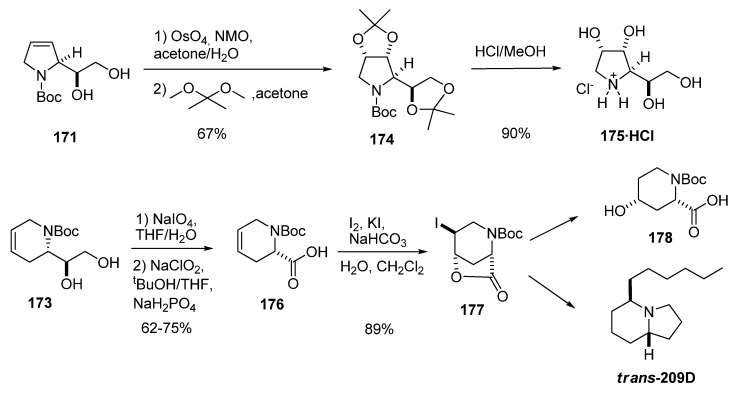
Synthetic applications of cyclic aminodiols (Riera *et al.* [64,87]).

Both intermediates were transformed into biologically active compounds (Scheme 33): **171** was converted into several polyhydroxylated pyrrolidines such as 1,4-dideoxy-1,4-imino-D-allitol (**175**), whereas enantiomerically pure **173** was transformed, *via* oxidation to baikianine **176**, into 4-hydroxy *N*-Boc-*cis*-4-hydroxypipecolic acid (**178**) [64] and the indolizidine alkaloid ***trans*-209 **(isolated from the skin of certain neotropical frogs) [87]. 

**Scheme 34:**
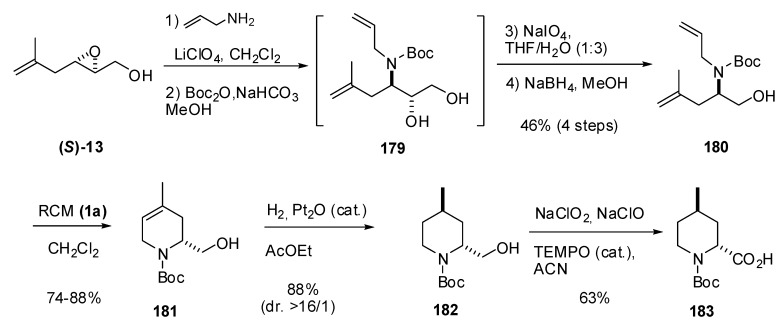
Estereoselective synthesis of *trans*-4-methylpipecolic acid **183 **(Riera *et al.* [88]).

The same methodology was used in the synthesis of *trans*-4-methylpipecolic acid (**183**), a fragment of the thrombin inhibitor argatroban [88]. In this case, the regioselectivity of the C3 ring-opening in the epoxide **(*S*)-13 **was somewhat lower than in **(*R*)-8** due to the increased steric hindrance of the side chain in the former (Scheme 34). However, oxidation with sodium perchlorate, followed by reduction of the aldehyde to the alcohol, enabled preparation of the diene **180** in good yield [46% from (***S***)-**13**)]. Subsequent RCM afforded dehydropiperidine **181** that was diastereoselectively hydrogenated to the *trans* isomer **182**. The primary alcohol was required for obtaining good diastereoselectivities in the hydrogenation. Oxidation of the alcohol to the acid enabled completion of the first asymmetric synthesis of *trans*-4-methylpipecolic acid (**183**).

**Scheme 35:**
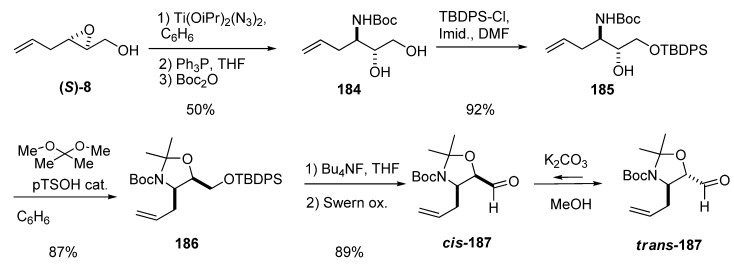
Preparation of carbaldehydes ***cis*** and ***trans*-187**, key intermediates in the synthesis of 3-amino-2,3,6-trideoxysugars, conduramines and aminocyclitols.

Ring-opening of epoxy alcohols at C3 with azide can provide excellent regioselectivity if performed with titanium diazidodiisopropoxide, whether isolated or prepared *in situ*. Riera *et al.* [89] used this reagent in a multigram synthesis of the *N*-Boc-3-amino-1,2-diol **184** that did not require purification of the intermediates. The epoxide **(*S*)-8** was treated with titanium diazidodiisopropoxide at 75 ºC in benzene, and the resulting crude azidodiol was reduced with triphenylphosphine and then protected with Boc_2_O, affording the diol **184** in 50% overall yield (Scheme 35). Diol **184** was converted into carbaldehydes **187** through a high yielding protocol of protecting group manipulation and Swern oxidation. These carbaldehydes were later used in the enantioselective synthesis of 3-amino-2,3,6-trideoxysugars, conduramines and aminocyclitols.

The 3-amino-2,3,6-trideoxysugars were prepared from carbaldehydes ***cis*-187** and ***trans*-187 **via diastereoselective addition of a methyl metal reagent followed by ozonolysis of the double bond [89]. Interestingly, in the reactions of ***cis*-187**, lithium dimethyl cuprate mainly afforded the *syn* alcohol (93:7) ***cis*-*syn*-188**, whereas MeLi/TiCl_4_ led primarily to the *anti* alcohol (96:4) ***cis*-*anti*-188**. Reductive ozonolysis followed by acetylation afforded protected D-daunosamine (**189**) and L-ristosamine (**190**). A similar trend was observed for ***trans-*187**, which was converted into the protected L-epi-daunosamine and D-acosamine using the same reaction sequence. Thus, this approach constitutes a general synthesis of 3-amino-2,3,6-trideoxysugars with complete control of the stereochemistry of the three contiguous stereogenic centers. 

**Scheme 36:**
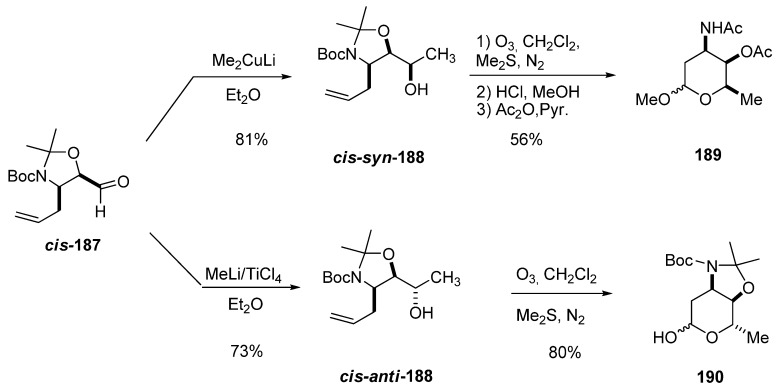
Synthesis of protected D-daunosamine (**189**) and L-ristosamine (**190**) from aldehyde ***cis*-187 **(Riera *et al. *[89]).

Diastereoselective addition of a vinyl group either to aldehydes ***cis ***and ***trans*-187** enabled preparation of dienes suitable for RCM. This strategy was used to prepare 4-deoxy-3-conduramines with full stereochemical control of three chiral centers [90]. As in the addition of the methyl group, the addition of lithium divinyl cuprate to ***cis*-187 **gave excellent stereoselectivity (95:1) towards the *syn* isomer ***cis-syn*-191**. Vinyl lithium afforded the *anti* isomer ***cis-anti*-191 **with less selectivity (4:1). RCM with first-generation Grubbs’ catalyst (**1a)** of dienes **191** afforded deoxyconduramines **192** in excellent yields. All isomers of the deoxyconduramines could be selectively prepared by choosing the enantiomer of the epoxide, the *cis*/*trans* stereochemistry of the aldehyde, and the organometallic vinyl reagent. Moreover, diastereoselective dihydroxylation provided a new family of aminocyclitols **193** with full stereochemical control. 

**Scheme 37:**
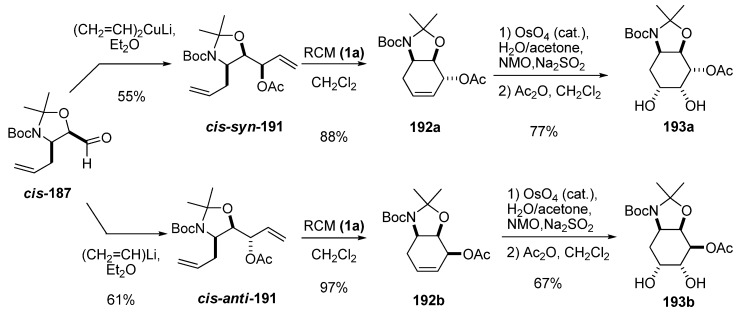
Syntheses of some isomers of deoxyconduramines and aminocyclitols from carbaldehyde ***cis*-187 **(Riera *et al.* [90]).

## 6. Conclusions

Epoxy alcohols with unsaturated side chains have been extensively applied to syntheses of biologically active compounds. They can be prepared in any absolute configuration by Sharpless asymmetric epoxidation. These epoxides have a rich chemistry of completely stereospecific transformations that yield products with predictable stereochemistry. Furthermore, the unsaturated side chain can be efficiently transformed via oxidation or olefin metatheses.
